# Using the theory of planned behavior to identify predictors of handwashing among Iranian healthcare workers

**DOI:** 10.1186/1753-6561-5-S6-P108

**Published:** 2011-06-29

**Authors:** M Askarian, N Maharlouei, F Yousefi, M-L Mclaws

**Affiliations:** 1 Community Medicine, Shiraz University of Medical Sciences, Shiraz, Iran, Islamic Republic Of; 2 Shiraz University, Shiraz, Iran, Islamic Republic Of; 3 Public Health & Community Medicine, UNSW, Sydney, Australia

## Introduction / objectives

The incidence of healthcare associated infections in a surgical ward in Shiraz has beenÂ established as 18%Â with associated costs of approx US150,000. Healthcare workers (HCWs) had acceptable levels of knowledge and attitude about hand hygiene but poor self-reported practices. We used the Theory of Planned Behavior (TPB) to identify predictors of handwashing to underpin a theory-driven intervention.

## Methods

Between April and September 2008, 1700 healthcare workers (HCWs) from all wards in 18 private and 10 public hospitals in Shiraz answered a self-administered survey designed in accordance with the TPB. Multiple logistic regression analysis was used to model two handwashing for patient contacts perceived to be clean and contacts perceived to contaminate hands.

## Results

90% of HCWs returned a completed survey. SignificantÂ predictors for clean contact handwashing in the hospital included compliance withÂ similar community practice (AOR2.1, P<0.000) and contaminated contact handwashingÂ (AOR1.6, P<0.000), perception that clean contact handwashingÂ required little effort (AOR1.1, P=0.039) and nursing peer pressure (AOR 1.1, P=0.025). SignificantÂ predictors for contaminated contact handwashing included clean contact handwashing compliance (AOR2.5, P<0.000), community contaminated contact handwashing (AOR1.5 P**=**0.001), peer pressure from ICPs (AOR1.4, P=0.001) and attitudes about contaminated contact handwashingÂ (AOR1.1, P=0.001).

## Conclusion

Community-based handwashing practices exert strong influence on handwashing in the hospital. Given the interdependence between community and hospital handwashing a campaign to improve aware about the benefit of community handwashing may improve HCWs’ compliance.

## Disclosure of interest

None declared.

